# Association between Obesity, Surgical Route, and Perioperative Outcomes in Patients with Uterine Cancer

**DOI:** 10.1155/2018/5130856

**Published:** 2018-06-19

**Authors:** Entidhar Al Sawah, Jason L. Salemi, Mitchel Hoffman, Anthony N. Imudia, Emad Mikhail

**Affiliations:** ^1^Department of Obstetrics and Gynecology, University of South Florida Morsani College of Medicine, Tampa, FL, USA; ^2^Department of Family and Community Medicine, Baylor College of Medicine, Houston, TX, USA; ^3^Division of Gynecologic Oncology, Moffitt Cancer Center, University of South Florida, Tampa, FL, USA

## Abstract

**Objective:**

To study temporal trends of hysterectomy routes performed for uterine cancer and their associations with body mass index (BMI) and perioperative morbidity.

**Methods:**

A retrospective review of the American College of Surgeons-National Surgical Quality Improvement Program (ACS-NSQIP) 2005-2013 databases was conducted. All patients who were 18 years old and older with a diagnosis of uterine cancer and underwent hysterectomy were identified using ICD-9-CM and CPT codes. Surgical route was classified into four groups: total abdominal hysterectomy (TAH), total vaginal hysterectomy (TVH), laparoscopic assisted vaginal hysterectomy (LAVH), and total laparoscopic hysterectomy (TLH) including both conventional and robotically assisted. Patients were then stratified according to BMI.

**Results:**

7199 records were included in the study. TLH was the most commonly performed route of hysterectomy regardless of BMI, with proportions of 50.9%, 48.9%, 50.4%, and 51.2% in ideal, overweight, obese, and morbidly obese patients, respectively. The median operative time for TAH was 2.2 hours compared to 2.7 hours for TLH (*p* < 0.01). The median length of stay for TAH was 3 days compared to 1 day for TLH (*p* < 0.01). The percentage of patients with an adverse outcome (composite indicator including transfusion, deep venous thrombosis, and infection) was 17.1 versus 3.7 for TAH and TLH, respectively (*p* < 0.01).

**Conclusion:**

During the last decade, TLH has been increasingly performed in women with uterine cancer. The increased adoption of TLH was seen in all BMI subgroups.

## 1. Introduction

Uterine cancer is the most common gynecologic cancer in USA [1], with the median age at presentation being 60 years [2]. Depending on the stage and grade, surgery is the mainstay of treatment of uterine tumors, with or without subsequent radiation. Early stage uterine cancer can be managed safely with conventional as well as robotically assisted laparoscopic approaches [3, 4].

Despite the evidence-based benefits associated with minimally invasive gynecologic surgical approaches, laparotomy remains the route of choice in more than 60% of the 600,000 hysterectomy procedures performed annually in USA [5]. The rate of abdominal hysterectomy in USA between 2003 and 2005 was still over 60% and only 12–14% of hysterectomies were being performed laparoscopically [6].

Greater degree of obesity is a well-known risk factor for the development of uterine cancer [7, 8]. Surgery for uterine cancer in obese patients can pose significant intra- and postoperative challenges to the surgeon. Obesity is associated with a higher rate of conversion of laparoscopic surgery to laparotomy and a lower completion rate of lymph node dissection [9].

Although obesity is associated with higher incidence of uterine cancer and recent studies have reported that the rate of abdominal hysterectomy performed for benign indications is increased in obese patients [10], the extent to which obesity plays a role in the surgical management of uterine cancer is currently unknown. We hypothesize that, with increased obesity, the proportion of minimally invasive surgical approach might decrease. Therefore, in this study, we explore the association between obesity and surgical route for the treatment of uterine cancer, and we describe the extent to which the rate of perioperative complications differs by obesity status.

## 2. Materials and Methods

After obtaining exempt status from the University of South Florida's institutional review board, we used the American College of Surgeons-National Surgical Quality Improvement Program (ACS NSQIP) database from 2005 to 2013 to conduct a retrospective, cross-sectional analysis of female patients with uterine cancer who underwent hysterectomy. ACS-NSQIP is a publically available and deidentified database created as part of a quality improvement initiative originally developed by the Veterans' Health Administration in 1991 and adopted by the American College of Surgeons in 2001 [11, 12]. The database includes more than 450 participating community and academic hospitals nationwide. Data captured include but are not limited to demographics, comorbidities, laboratory values, and operative variables, as well as 30-day postoperative outcomes, complications, mortality, reoperation, and length of stay. Quality improvement and assurance protocols include routine auditing and the use of specially trained surgical nurses to record patient variables. A random 8-day sampling method is used to ensure that a diverse range of surgical procedures is captured.

**Figure 1:**
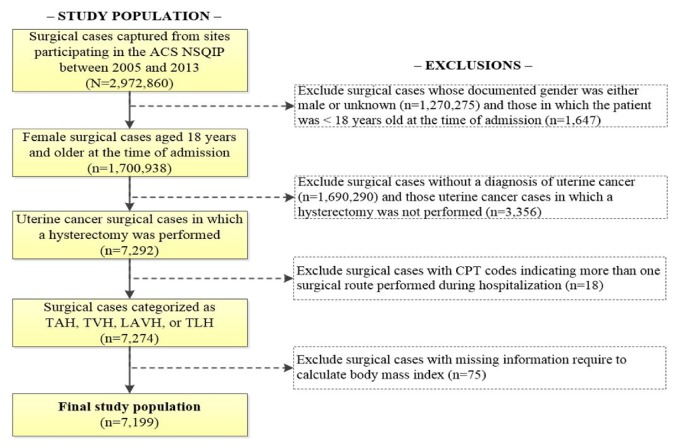
**Flow diagram representing the final determination of all patients in which a hysterectomy was performed among patients with a diagnosis of uterine cancer, ACS-NSQIP, 2005-2013**. ACS-NSQIP: American College of Surgeons-National Surgical Quality Improvement Program; TAH: total abdominal hysterectomy; TVH: total vaginal hysterectomy; LAVH: laparoscopic assisted vaginal hysterectomy; TLH: total laparoscopic hysterectomy.

In patients aged 18 years or above, we identified women with uterine cancer using the principal postoperative diagnosis (ICD-9-CM code 182.0, 182.1, or 182.8). We then used current procedural terminology (CPT) procedure codes to identify 7,292 surgical cases in which hysterectomy was performed and then specific codes were used to subclassify cases as (1) total abdominal hysterectomy (TAH, 58150 and 58200); (2) total vaginal hysterectomy (TVH, 58260, 58262, 58263, 58270, 58275, 58280, 58290, 58291, 58292, and 58294); (3) laparoscopic assisted vaginal hysterectomy (LAVH, 58550, 58552, 58553, and 58554); and (4) total laparoscopic hysterectomy (TLH, 58570, 58571, 58572, and 58573). We excluded 18 cases (0.2%) with CPT codes indicating more than one surgical route performed. Due to absence of specific CPT codes, we could not differentiate between robotically assisted laparoscopic hysterectomies from TLH; therefore, these groups are combined into a single group (TLH) (Figure 1).

Body mass index (BMI) was calculated as [(weight in pounds)/((height in inches)^2^) × 703], and then patients were classified according to BMI as follows: normal and underweight (<25), overweight (25–29.9), obese (classes I and II; 30–39.9), and morbid obesity (class III; ≥40). We excluded 75 cases (1.0%) for which BMI could not be calculated due to missing information on presurgical weight and height. Data analyzed included patient age, race/ethnicity, operative time, length of hospital stay, blood transfusion, development of deep venous thrombosis, and development of surgical infection. Infection types included superficial surgical site (involving only skin or subcutaneous tissue) and deep incisional surgical site (involving deep soft tissues). When data were available (2011 or later), we also captured 30-day readmissions.

Descriptive statistics were used to describe the frequency and temporal trends in surgical approaches in the entire study population and stratified by patient's BMI level. Differences in the distribution of selected patient sociodemographic and hospital characteristics and in the rates of clinical outcomes by surgical approach were assessed using either a Wilcoxon-Mann–Whitney test (continuous variables) or chi-square test (categorical variables). For each hysterectomy route, we compared the rate of perioperative outcomes across levels of patient's BMI. All statistical analyses were conducted using SAS version 9.4 (SAS Institute, Inc., Cary, NC), using a 5% type I error rate and two-sided hypothesis tests. STrengthening the Reporting of OBservational studies in Epidemiology (STROBE) guidelines for reporting observational studies were followed for this study [13].

## 3. Results

**Figure 2:**
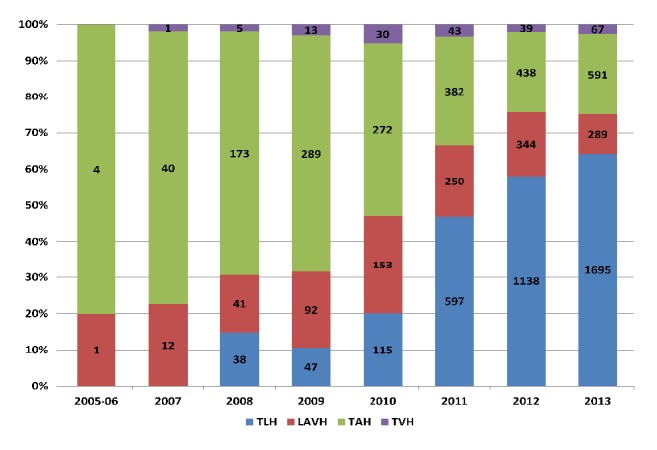
**Frequency and proportion of different types of hysterectomies performed among patients with a diagnosis of uterine cancer, ACS-NSQIP, 2005-2013**. TAH: total abdominal hysterectomy; TVH: total vaginal hysterectomy; LAVH: laparoscopic assisted vaginal hysterectomy; TLH: total laparoscopic hysterectomy.

Between 2005 and 2013, we identified a total of 7,199 uterine cancer surgical cases managed with hysterectomy and with documented presurgical weight and height (Figure 1). The most common route of hysterectomy was TLH (50.4%), followed by TAH (30.4%), LAVH (16.4%), and TVH (2.8%). During the study period, we observed a relative increase in the use of the TLH route (from 15% in 2008 to 64% in 2013) and a concomitant relative decrease in the use of TAH (from 67% in 2008 to 22% in 2013) (Figure 2). Until 2013, the proportion of hysterectomies using the LAVH route was relatively constant over time, as was the small proportion of surgical cases in which TVH was performed.

**Figure 3:**
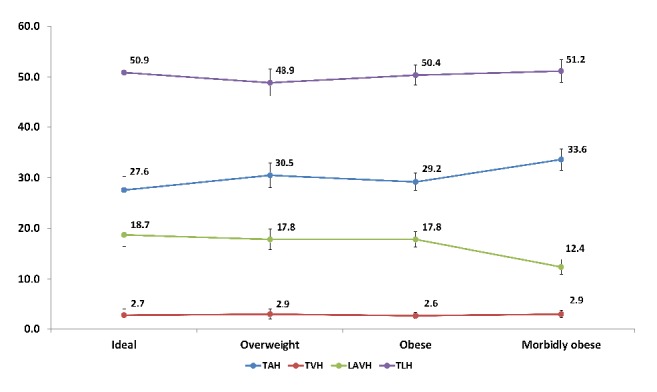
Rates of different types of hysterectomy performed among patients with a diagnosis of uterine cancer, by patient's body mass index, ACS-NSQIP, 2005-2013.

Total laparoscopic hysterectomy was the most performed procedure regardless of BMI, occurring in 50.9%, 48.9%, 50.4%, and 51.2% of patients with ideal, overweight, obese, and morbidly obese BMI, respectively (Figure 3). The overall rate of TAH was 30%; however, the rate tended to be slightly higher in patients who were morbidly obese (33.6%) relative to other patients. This increase in the rate of TAH in morbidly obese patients was at the expense of LAVH (12.4%); the LAVH rate among the morbidly obese was statistically significantly lower than the LAVH rate in each of the other BMI categories.

The median patient BMI differed significantly by surgical approach; patients who underwent TAH had overall higher BMI (34.0 kg/m^2^) compared to those undergoing TVH (33.1 kg/m^2^), LAVH (32.2 kg/m^2^), and TLH (33.7 kg/m^2^) (Table 1). Patients undergoing TAH and LAVH were more likely to be nonwhite than patients undergoing TVH and TLH (*p* < 0.01). The median operative room time was statistically significantly shorter for TAH (2.2 hours) compared to TLH (2.7 hours). However, the median length of hospital stay for TAH was three times longer than TLH, TVH, or LAVH (*p* < 0.01). Over 15% of patients who underwent TAH stayed in the hospital for 6 days or longer, compared to only 1.3% of patients undergoing TLH (*p* < 0.01). The rates of several perioperative complications were increased significantly in patients who underwent TAH compared to TLH; these complications include transfusion (10.3% versus 1.7%) (*p* < 0.01), surgical site infection (7.1% versus 1.7%) (*p* < 0.01), and readmission within 30 days (8.9% versus 3.8%) (*p* < 0.01).

Regardless of the route of hysterectomy, patients who were morbidly obese, obese, or overweight tended to have statistically significantly longer operation times than patients who had an ideal BMI (*p* < 0.05). Similarly, postoperative infections, including superficial or deep surgical site infections, were more common in higher BMI categories when compared with ideal BMI (*p* < 0.05), and patients who were morbidly obese experienced substantially higher rates of any surgical site or wound infection (6.4%) compared to patients who had an ideal BMI (1.6%). The 30-day readmission rates were similar across all BMI categories (Table 2).

The rate of readmission and the rate of a composite infection outcome (including superficial surgical site infection, open wound/wound infection, and deep incisional surgical site infection) within 30 days were significantly lower for TLH and LAVH compared to TAH in all BMI subgroups except for the overweight group (Supplemental Tables S1-S4).

In comparing perioperative outcomes of TLH stratified by patient's BMI category, it was noted that increasing degree of obesity was associated with longer operative time. The mean operation time in patients of ideal weight (145 min) was shorter compared to overweight, obese, and morbidly obese women (159, 158, and 171 minutes, resp., *p* < 0.05). Also, the rate of open surgical wound or wound infection was higher in the morbidly obese group (1.3%) compared to ideal weight patients (0.2%) (*p* < 0.05). All other perioperative outcomes were not statistically significantly different across BMI levels (Table 3).

## 4. Discussion

In this study, using the ACS-NSQIP database, TLH (including conventional or robotically assisted) was found to be the most frequently chosen route for hysterectomy for surgical management of patients with uterine cancer. Performance of TLH increased from 16.5% in 2008 to 64.1% in 2013. We also found that TLH is the most commonly chosen route regardless of the degree of obesity. Despite increased operative time compared to abdominal hysterectomy, the minimally invasive approach provided better perioperative outcomes manifesting in decreased length of hospital stay and decreased rates of transfusion, surgical site infection, and readmission within 30 days. The utilization of TLH was not negatively impacted by the degree of obesity, despite the increase in operative time and surgical infection.

The results from current study are consistent with data from the SEER database, which showed that performance of minimally invasive hysterectomy has increased from 9.3% in 2006 to 61.7% in 2011 [14]. Minimally invasive surgery improved outcomes including decreased hospital stay, increased patient quality of life, consistency with patient preference, and enhanced cosmesis [15]. These factors have been considered as important drivers of cost and efficiency in the era of the Affordable Care Act [16].

Compared to other routes, TAH rates among patients with uterine cancer were higher in morbidly obese women compared to women with lower BMI levels. This might be explained by the increased and persistent technical challenges encountered by surgeons during minimally invasive surgery in patients with higher degrees of obesity [17]. Excessive adiposity poses several challenges to the surgical team, including poor patient tolerance to Trendelenburg positioning and positive intra-abdominal pressure, surgeons' fatigue, and the inability to correctly expose and develop the anatomical spaces [18].

On the other hand, TAH was found to be associated with an increased risk for perioperative complications when compared with other surgical routes. This is reflected by longer hospital stay, higher rates of surgical sites infections, and higher 30-day readmission rates. Since higher perioperative complications can compromise overall survival and success of adjuvant therapy, it is critical to take active measures to avoid or reduce the incidence of such complications [19]. With accumulating experience and increased training in minimally invasive surgeries, including robotically assisted procedures, the adoption of such techniques is likely to increase in the future; we suspect a concomitant improvement in perioperative outcomes in patients with uterine cancer [5].

In cases where pelvic lymph node dissection is performed, the preferred route for hysterectomy is either abdominal or laparoscopic. The utilization of TVH for uterine cancer surgery is controversial and the utility of nodal dissection in uterine cancer patients lacks a consensus opinion [20]. The role of TVH in uterine cancer depends on the type of the tumor, the stage of tumor, BMI, and presence of comorbidities. For stage I grade I uterine cancer, TVH may be reasonable, especially if CA-125 level <20 U/mL because of the low likelihood of extrauterine tumor invasion [21]. TVH utilization is limited in more advanced uterine cancer due to the limited ability to complete cancer staging.

In comparison with higher degrees of obesity, ideal body weight was found to be associated with the most favorable perioperative outcomes. This is supported by other studies showing obesity to be associated with increased complication rates in elective hysterectomy procedures, independent of the surgical route. Morbid obesity was found to be associated with increased conversion of laparoscopic surgery to laparotomy and less complete lymph node dissection [19, 22, 23]. Obesity is now considered as a pandemic with increasing prevalence [24]. It has been shown that patients who are obese experience some of the greatest differential benefits from minimally invasive techniques [10]. Obesity increased the risk of unintended conversion to laparotomy, where patients with BMI >40 have 4-fold increase in the conversion rate [18].

The ACS-NSQIP database that was used in this study represents a major strength due to its multi-institutional nature and is widely considered to be accurate, reproducible, and reliable. Data are collected by specially trained surgical clinical nurse reviewers who collect more than 100 clinical variables, including preoperative risk factors, intraoperative variables, and 30-day postoperative mortality and morbidity outcomes for patients undergoing major surgical procedures [25].

A weakness of this study is its observational nature. Although clinical trials can be the best research path to delineate optimum surgical approach for uterine cancer in morbidly obese patients, observational studies can be invaluable tool for hypothesis generation and prediction of patients who are at higher risk of complication of a certain therapeutic approach. Another weakness of the study is the fact that it lacked data on patient survival and its association with BMI categories and the inability to differentiate between conventional laparoscopic and robotic procedures. The data in the ACS-NSQIP are only from participating hospitals and, despite being distributed throughout USA, they do not collectively represent a statistically selected nationally representative sample. This study also lacks data regarding lymph node dissection; traditionally obesity is thought to be associated with less complete lymph node dissection; interestingly, in a study by Uccella et al, it was found that the number of lymph nodes removed was not affected by BMI [18]. TLH is currently the most commonly performed route for hysterectomy for patients with uterine cancer, regardless of the degree of obesity. Other confounding variables including surgical experience, hospital to hospital variation, and ethnicity could not be controlled for in this analysis.

Obesity poses an important challenge for the surgeon in selecting the surgical modality that balances between the technical difficultly and obtaining the best perioperative surgical outcomes.

## Figures and Tables

**Table 1:** Distribution of selected patient sociodemographic and hospital characteristics among patients with a diagnosis of uterine cancer and in which a hysterectomy was performed, by route of hysterectomy, ACS-NSQIP, 2005-2013.

	**TAH** **(n=2,189)**		**TVH** **(n=198)**		**LAVH** **(n=1,182)**		**TLH** **(n=3,630)**		***P*** ^**a**^
BMI (kg/m^2^), med (Q1-Q3)	34.0	28.0-42.2	33.1	27.8-41.6	32.2	26.6-38.7	33.7	27.4-41.2	<.01
BMI (kg/m^2^)									<.01
<25	313	14.3	31	15.7	212	17.9	577	15.9	
25-30	428	19.6	40	20.2	250	21.2	687	18.9	
30-40	776	35.4	70	35.4	472	39.9	1,341	36.9	
≥40	672	30.7	57	28.8	248	21.0	1,025	28.2	
Age at admission (years)									<.01
<50	260	11.9	23	11.6	138	11.7	358	9.9	
50-59	572	26.1	40	20.2	325	27.5	1,029	28.3	
60-69	741	33.9	68	34.3	411	34.8	1,354	37.3	
70-79	284	17.5	30	15.2	214	18.1	655	18.0	
≥80	232	10.6	37	18.7	94	8.0	234	6.4	
Race/ethnicity									<.01
Non-Hispanic white	1,426	65.1	162	81.8	790	66.8	2,939	81.0	
Non-Hispanic black	228	10.4	4	2.0	85	7.2	187	5.2	
Hispanic	140	6.4	9	4.5	95	8.0	157	4.3	
Other	82	3.7	12	6.1	49	4.1	190	5.2	
Missing	313	14.3	11	5.6	163	13.8	157	4.3	
Total operation time (hours), med (Q1-Q3)	2.2	1.6-3.0	1.6	1.1-2.8	2.6	1.9-3.6	2.7	2.0-3.5	<.01
Total operation time (hours)									<.01
<2	928	42.4	125	63.1	320	27.1	857	23.6	
2-3	700	32.0	29	14.6	394	33.3	1,374	37.9	
3-4	369	16.9	20	10.1	245	20.7	842	23.2	
4-5	115	5.3	14	7.1	144	12.2	371	10.2	
≥5	77	3.5	10	5.1	79	6.7	184	5.1	
Length of stay (days), med (Q1-Q3)	3.0	2.0-4.0	1.0	1.0-2.0	1.0	1.0-2.0	1.0	1.0-1.0	<.01
Length of stay (days)									<.01
0-1	97	4.4	140	70.7	833	70.5	2,920	80.4	
2	484	22.1	44	22.2	240	20.3	467	12.9	
3-5	1,271	58.1	11	5.6	90	7.6	193	5.3	
≥6	336	15.3	3	1.5	19	1.6	49	1.3	
Bleeding transfusion^b^	225	10.3	7	3.5	29	2.5	60	1.7	<.01
DVT/thrombophlebitis	20	0.9	3	1.5	6	0.5	17	0.5	0.07
Superficial surgical site infection^c^	107	4.9	2	1.0	3	0.3	33	0.9	<.01
Open wound/wound infection^d^	20	0.9	2	1.0	4	0.3	22	0.6	0.21
Deep incisional surgical site infection^e^	31	1.4	0	0.0	0	0.0	8	0.2	<.01
Any infection listed above	156	7.1	4	2.0	7	0.6	63	1.7	<.01
Any adverse outcome listed above	375	17.1	13	6.6	41	3.5	135	3.7	<.01
Readmission within 30 days^f^	125	8.9	8	5.4	30	3.4	129	3.8	<.01

ACS-NSQIP: American College of Surgeons-National Surgical Quality Improvement Program; BMI: body mass index; DVT: deep vein thrombosis.

Unless otherwise indicated, values listed are the frequency and percent.

^a^P value from either a Wilcoxon-Mann–Whitney test (continuous variables) or chi-square test (categorical variables).

^b^At least 1 unit of packed or whole red blood cells given from the surgical start time up to and including 72 hours postoperatively.

^c^Infection that occurs within 30 days after the operation and the infection involves only skin or subcutaneous tissue of the incision.

^d^Preoperative evidence of a documented open wound at the time of the principal operative procedure. An open wound is a breach in the integrity of the skin or separation of skin edges and includes open surgical wounds, with or without cellulitis or purulent exudate. This does not include osteomyelitis or localized abscesses.

^e^Infection that occurs within 30 days after the operation and the infection appears to be related to the operation and infection involved deep soft tissues (e.g., fascial and muscle layers) of the incision.

^f^Readmission within 30 days was only available beginning in 2011; therefore, the percent provided reflects only the proportion of 2011-13 cases who were readmitted.

**Table 2:** Perioperative outcomes stratified by patient's body mass index, ACS-NSQIP, 2005-2013.

	**BMI classification**			
**Characteristic/outcome**	**Ideal**	**Overweight**	**Obese**	**Morbidly obese**
Patient age (years)^a^	63 (55-74)	65 (57-73)	63 (57-71)	60 (54-65)*∗*
Operation time (min)^a^	137 (100-189)	143 (102-193)*∗*	149 (110-200)*∗*	164 (124-214)*∗*
Length of hospital stay (days)^a^	1 (1-2)	1 (1-3)	1 (1-3)	1 (1-3)*∗*
Bleeding transfusion^b^	79 (7.0)	70 (5.0)*∗*	90 (3.4)*∗*	82 (4.1)*∗*
DVT/thrombophlebitis	3 (0.3)	11 (0.8)	18 (0.7)	14 (0.7)
Superficial surgical site infection^c^	11 (1.0)	10 (0.7)	53 (2.0)*∗*	71 (3.5)*∗*
Open wound/wound infection^d^	5 (0.4)	4 (0.3)	10 (0.4)	29 (1.4)*∗*
Deep incisional surgical site infection^e^	2 (0.2)	2 (0.1)	7 (0.3)	28 (1.4)*∗*
Any infection listed above	18 (1.6)	16 (1.1)	68 (2.6)	128 (6.4)*∗*
Any adverse outcome listed above	96 (8.5)	91 (6.5)	167 (6.3)*∗*	210 (10.5)
Readmission within 30 days^f^	46 (5.0)	47 (4.1)	101 (4.6)	98 (6.0)

ACS NSQIP = American College of Surgeons National Surgical Quality Improvement Program; BMI = body mass index; DVT = deep vein thrombosis.

*∗*P-value<0.05 from either a Wilcoxon-Mann Whitney test (continuous variables) or chi-square test (categorical variables). For each outcome, three tests are performed: overweight vs. ideal BMI, obese vs. ideal BMI, morbidly obese vs. ideal BMI.

^a^Values presented as median (Q1-Q3); all others are presented as frequency (%).

^b^At least 1 unit of packed or whole red blood cells given from the surgical start time up to and including 72 hours postoperatively.

^c^Infection that occurs within 30 days after the operation and the infection involves only skin or subcutaneous tissue of the incision.

^d^Preoperative evidence of a documented open wound at the time of the principal operative procedure. An open wound is a breach in the integrity of the skin or separation of skin edges and includes open surgical wounds, with or without cellulitis or purulent exudate. This does not include osteomyelitis or localized abscesses.

^e^Infection that occurs within 30 days after the operation and the infection appears to be related to the operation and infection involved deep soft tissues (e.g., fascial and muscle layers) of the incision.

^f^Readmission within 30 days was only available beginning in 2011; therefore, the percent provided reflects only the proportion of 2011-13 cases who were readmitted.

**Table 3:** Perioperative outcomes among uterine cancer patients undergoing total laparoscopic hysterectomy, stratified by patient's body mass index, ACS-NSQIP, 2005-2013.

	**BMI classification**			
**Characteristic/outcome**	**Ideal**	**Overweight**	**Obese**	**Morbidly obese**
Patient age (years)^a^	63 (55-72)	65 (57-72)*∗*	63 (57-70)	60 (54-66)*∗*
Operation time (min)^a^	145 (113-194)	159 (119-205)*∗*	158 (120-206)*∗*	171 (134-218)*∗*
Length of hospital stay (days)^a^	1 (1-1)	1 (1-1)	1 (1-1)	1 (1-1)*∗*
Bleeding transfusion^b^	16 (2.8)	12 (1.7)	18 (1.3)*∗*	14 (1.4)
DVT/thrombophlebitis	0 (0.0)	4 (0.6)	6 (0.4)	7 (0.7)
Superficial surgical site infection^c^	3 (0.5)	4 (0.6)	13 (1.0)	13 (1.3)
Open wound/wound infection^d^	1 (0.2)	1 (0.1)	7 (0.5)	13 (1.3)*∗*
Deep incisional surgical site infection^e^	0 (0.0)	1 (0.1)	2 (0.1)	5 (0.5)
Any infection listed above	4 (0.7)	6 (0.9)	22 (1.6)	31 (3.0)*∗*
Any adverse outcome listed above	20 (3.5)	20 (2.9)	44 (3.3)	51 (5.0)
Readmission within 30 days^f^	15 (2.8)	23 (3.6)	50 (3.9)	41 (4.2)

ACS NSQIP = American College of Surgeons National Surgical Quality Improvement Program; BMI = body mass index; DVT = deep vein thrombosis.

*∗*P-value<0.05 from either a Wilcoxon-Mann Whitney test (continuous variables) or chi-square test (categorical variables). For each outcome, three tests are performed: overweight vs. ideal BMI, obese vs. ideal BMI, morbidly obese vs. ideal BMI.

^a^Values presented as median (Q1-Q3); all others are presented as frequency (%).

^b^At least 1 unit of packed or whole red blood cells given from the surgical start time up to and including 72 hours postoperatively.

^c^Infection that occurs within 30 days after the operation and the infection involves only skin or subcutaneous tissue of the incision.

^d^Preoperative evidence of a documented open wound at the time of the principal operative procedure. An open wound is a breach in the integrity of the skin or separation of skin edges and includes open surgical wounds, with or without cellulitis or purulent exudate. This does not include osteomyelitis or localized abscesses.

^e^Infection that occurs within 30 days after the operation and the infection appears to be related to the operation and infection involved deep soft tissues (e.g., fascial and muscle layers) of the incision.

^f^Readmission within 30 days was only available beginning in 2011; therefore, the percent provided reflects only the proportion of 2011-13 cases who were readmitted.

## Data Availability

Data are available upon request.

## Abbreviations

BMI: Body mass index
ACS-NSQIP: American College of Surgeons-National Surgical Quality Improvement Program
CPT: Current procedural terminology
ICD-9-CM: International Classification of Disease, Ninth Revision, Clinical Modification
TAH: Total abdominal hysterectomy
TVH: Total vaginal hysterectomy
LAVH: Laparoscopic assisted vaginal hysterectomy
TLH: Total laparoscopic hysterectomy.

## Supplementary Materials

Supplementary Materials Table S1: perioperative outcomes among uterine cancer patients with ideal BMI, stratified by route of hysterectomy, ACS-NSQIP, 2005-2013. Table S2: perioperative outcomes among uterine cancer patients with overweight BMI, stratified by route of hysterectomy, ACS-NSQIP, 2005-2013. Table S3: perioperative outcomes among uterine cancer patients with obese BMI, stratified by route of hysterectomy, ACS-NSQIP, 2005-2013. Table S4: perioperative outcomes among uterine cancer patients with morbidly obese BMI, stratified by route of hysterectomy, ACS-NSQIP, 2005-2013.
