# Supplementary material for: Association between Obesity, Surgical Route, and Perioperative Outcomes in Patients with Uterine Cancer
Source: Minim Invasive Surg. 2018 Jun 19;2018:5130856. doi: 10.1155/2018/5130856 (PMC6029454; doi:10.1155/2018/5130856)
Supplement: Supplementary Materials — Table S1: perioperative outcomes among uterine cancer patients with ideal BMI, stratified by route of hysterectomy, ACS-NSQIP, 2005-2013. Table S2: perioperative outcomes among uterine cancer patients with overweight BMI, stratified by route of hysterectomy, ACS-NSQIP, 2005-2013. Table S3: perioperative outcomes among uterine cancer patients with obese BMI, stratified by route of hysterectomy, ACS-NSQIP, 2005-2013. Table S4: perioperative outcomes among uterine cancer patients with morbidly obese BMI, stratified by route of hysterectomy, ACS-NSQIP, 2005-2013. [file 5130856.f1.doc]

**Supplemental Material**

| **Table S1. Perioperative outcomes among uterine cancer patients with ideal BMI, stratified by route of hysterectomy, ACS NSQIP, 2005-2013** | | | | |
| --- | --- | --- | --- | --- |
|  | **Hysterectomy Route** | | | |
| **Characteristic/outcome** | **TAH** | **TVH** | **LAVH** | **TLH** |
| Patient age (years) ^a^ | 65 (56-75) | 68 (55-81) | 65 (56-75) | 63 (55-72)* |
| Operation time (min) ^a^ | 109 (80-158) | 96 (58-175) | 148 (102-201)* | 145 (113-194)* |
| Length of hospital stay (days) ^a^ | 3 (2-5) | 1 (1-2)* | 1 (1-2)* | 1 (1-1)* |
| Bleeding transfusion^b^ | 53 (16.9) | 3 (9.7) | 7 (3.3)* | 16 (2.8)* |
| DVT/thrombophlebitis | 2 (0.6) | 1 (3.2) | 0 (0.0) | 0 (0.0) |
| Superficial surgical site infection^c^ | 7 (2.2) | 0 (0.0) | 1 (0.5) | 3 (0.5)* |
| Open wound/wound infection^d^ | 3 (1.0) | 0 (0.0) | 1 (0.5) | 1 (0.2) |
| Deep incisional surgical site infection^e^ | 2 (0.6) | 0 (0.0) | 0 (0.0) | 0 (0.0) |
| Any infection listed above | 12 (3.8) | 0 (0.0) | 2 (0.9)* | 4 (0.7)* |
| Any adverse outcome listed above | 63 (20.1) | 4 (12.9) | 9 (4.2)* | 20 (3.5)* |
| Readmission within 30 days^f^ | 23 (10.5) | 2 (10.5) | 6 (3.8)* | 15 (2.8)* |
| ACS NSQIP = American College of Surgeons National Surgical Quality Improvement Program; BMI = body mass index; DVT = deep vein thrombosis  *P-value<0.05 from either a Wilcoxon-Mann Whitney test (continuous variables) or chi-square test (categorical variables). For each outcome, three tests are performed: TVH vs. TAH, LAVH vs. TAH, TLH vs. TAH.  ^a^Values presented as median (Q1-Q3) ; all others are presented as frequency (%).  ^b^At least 1 unit of packed or whole red blood cells given from the surgical start time up to and including 72 hours postoperatively.  ^c^Infection that occurs within 30 days after the operation and the infection involves only skin or subcutaneous tissue of the incision.  ^d^Preoperative evidence of a documented open wound at the time of the principal operative procedure. An open wound is a breach in the integrity of the skin or separation of skin edges and includes open surgical wounds, with or without cellulitis or purulent exudate. This does not include osteomyelitis or localized abscesses.  ^e^Infection that occurs within 30 days after the operation and the infection appears to be related to the operation and infection involved deep soft tissues (e.g., fascial and muscle layers) of the incision.  ^f^Readmission within 30 days was only available beginning in 2011; therefore, the percent provided reflects only the proportion of 2011-13 cases who were readmitted. | | | | |

| **Table S2. Perioperative outcomes among uterine cancer patients with overweight BMI, stratified by route of hysterectomy, ACS NSQIP, 2005-2013** | | | | |
| --- | --- | --- | --- | --- |
|  | **Hysterectomy Route** | | | |
| **Characteristic/outcome** | **TAH** | **TVH** | **LAVH** | **TLH** |
| Patient age (years) ^a^ | 66 (58-76) | 76 (65-85)* | 63 (57-73)* | 65 (57-72)* |
| Operation time (min) ^a^ | 120 (89-168) | 87 (61-184)* | 147 (110-198)* | 159 (119-205)* |
| Length of hospital stay (days) ^a^ | 3 (2-5) | 1 (1-2)* | 1 (1-2)* | 1 (1-1)* |
| Bleeding transfusion^b^ | 53 (12.4) | 1 (2.5) | 4 (1.6)* | 12 (1.7)* |
| DVT/thrombophlebitis | 7 (1.6) | 0 (0.0) | 0 (0.0) | 4 (0.6) |
| Superficial surgical site infection^c^ | 6 (1.4) | 0 (0.0) | 0 (0.0) | 4 (0.6) |
| Open wound/wound infection^d^ | 2 (0.5) | 0 (0.0) | 1 (0.4) | 1 (0.1) |
| Deep incisional surgical site infection^e^ | 1 (0.2) | 0 (0.0) | 0 (0.0) | 1 (0.1) |
| Any infection listed above | 9 (2.1) | 0 (0.0) | 1 (0.4) | 6 (0.9) |
| Any adverse outcome listed above | 65 (15.2) | 1 (2.5)* | 5 (2.0)* | 20 (2.9)* |
| Readmission within 30 days^f^ | 18 (6.4) | 0 (0.0) | 6 (3.4) | 23 (3.6) |
| ACS NSQIP = American College of Surgeons National Surgical Quality Improvement Program; BMI = body mass index; DVT = deep vein thrombosis  *P-value<0.05 from either a Wilcoxon-Mann Whitney test (continuous variables) or chi-square test (categorical variables).  ^a^Values presented as median (Q1-Q3) ; all others are presented as frequency (%).  ^b^At least 1 unit of packed or whole red blood cells given from the surgical start time up to and including 72 hours postoperatively.  ^c^Infection that occurs within 30 days after the operation and the infection involves only skin or subcutaneous tissue of the incision.  ^d^Preoperative evidence of a documented open wound at the time of the principal operative procedure. An open wound is a breach in the integrity of the skin or separation of skin edges and includes open surgical wounds, with or without cellulitis or purulent exudate. This does not include osteomyelitis or localized abscesses.  ^e^Infection that occurs within 30 days after the operation and the infection appears to be related to the operation and infection involved deep soft tissues (e.g., fascial and muscle layers) of the incision.  ^f^Readmission within 30 days was only available beginning in 2011; therefore, the percent provided reflects only the proportion of 2011-13 cases who were readmitted. | | | | |

| **Table S3. Perioperative outcomes among uterine cancer patients with obese BMI, stratified by route of hysterectomy, ACS NSQIP, 2005-2013** | | | | |
| --- | --- | --- | --- | --- |
|  | **Hysterectomy Route** | | | |
| **Characteristic/outcome** | **TAH** | **TVH** | **LAVH** | **TLH** |
| Patient age (years) ^a^ | 64 (57-72) | 65 (58-74) | 62 (57-70) | 63 (57-70) |
| Operation time (min) ^a^ | 130 (96-182) | 102 (71-171)* | 157 (118-223)* | 158 (120-206)* |
| Length of hospital stay (days) ^a^ | 3 (2-4) | 1 (1-2)* | 1 (1-2)* | 1 (1-1)* |
| Bleeding transfusion^b^ | 61 (7.9) | 1 (1.4)* | 10 (2.1)* | 18 (1.3)* |
| DVT/thrombophlebitis | 6 (0.8) | 2 (2.9) | 4 (0.8) | 6 (0.4) |
| Superficial surgical site infection^c^ | 39 (5.0) | 1 (1.4) | 0 (0.0)* | 13 (1.0)* |
| Open wound/wound infection^d^ | 3 (0.4) | 0 (0.0) | 0 (0.0) | 7 (0.5) |
| Deep incisional surgical site infection^e^ | 5 (0.6) | 0 (0.0) | 0 (0.0) | 2 (0.1) |
| Any infection listed above | 45 (5.8) | 1 (1.4) | 0 (0.0)* | 22 (1.6)* |
| Any adverse outcome listed above | 105 (13.5) | 4 (5.7) | 14 (3.0)* | 44 (3.3)* |
| Readmission within 30 days^f^ | 33 (6.7) | 5 (9.6) | 13 (3.6)* | 50 (3.9)* |
| ACS NSQIP = American College of Surgeons National Surgical Quality Improvement Program; BMI = body mass index; DVT = deep vein thrombosis  *P-value<0.05 from either a Wilcoxon-Mann Whitney test (continuous variables) or chi-square test (categorical variables).  ^a^Values presented as median (Q1-Q3) ; all others are presented as frequency (%).  ^b^At least 1 unit of packed or whole red blood cells given from the surgical start time up to and including 72 hours postoperatively.  ^c^Infection that occurs within 30 days after the operation and the infection involves only skin or subcutaneous tissue of the incision.  ^d^Preoperative evidence of a documented open wound at the time of the principal operative procedure. An open wound is a breach in the integrity of the skin or separation of skin edges and includes open surgical wounds, with or without cellulitis or purulent exudate. This does not include osteomyelitis or localized abscesses.  ^e^Infection that occurs within 30 days after the operation and the infection appears to be related to the operation and infection involved deep soft tissues (e.g., fascial and muscle layers) of the incision.  ^f^Readmission within 30 days was only available beginning in 2011; therefore, the percent provided reflects only the proportion of 2011-13 cases who were readmitted. | | | | |

| **Table S4. Perioperative outcomes among uterine cancer patients with morbidly obese BMI, stratified by route of hysterectomy, ACS NSQIP, 2005-2013** | | | | |
| --- | --- | --- | --- | --- |
|  | **Hysterectomy Route** | | | |
| **Characteristic/outcome** | **TAH** | **TVH** | **LAVH** | **TLH** |
| Patient age (years) ^a^ | 59 (53-65) | 60 (53-67) | 60 (53-65) | 60 (54-66) |
| Operation time (min) ^a^ | 150 (112-195) | 105 (70-153)* | 177 (138-243)* | 171 (134-218)* |
| Length of hospital stay (days) ^a^ | 3 (3-5) | 1 (1-1)* | 1 (1-2)* | 1 (1-1)* |
| Bleeding transfusion^b^ | 58 (8.6) | 2 (3.5) | 8 (3.2)* | 14 (1.4)* |
| DVT/thrombophlebitis | 5 (0.7) | 0 (0.0) | 2 (0.8) | 7 (0.7) |
| Superficial surgical site infection^c^ | 55 (8.2) | 1 (1.8) | 2 (0.8)* | 13 (1.3)* |
| Open wound/wound infection^d^ | 12 (1.8) | 2 (3.5) | 2 (0.8) | 13 (1.3) |
| Deep incisional surgical site infection^e^ | 23 (3.4) | 0 (0.0) | 0 (0.0)* | 5 (0.5)* |
| Any infection listed above | 90 (13.4) | 3 (5.3) | 4 (1.6)* | 31 (3.0)* |
| Any adverse outcome listed above | 142 (21.1) | 4 (7.0)* | 13 (5.2)* | 51 (5.0)* |
| Readmission within 30 days^f^ | 51 (12.2) | 1 (2.2)* | 5 (2.7)* | 41 (4.2)* |
| ACS NSQIP = American College of Surgeons National Surgical Quality Improvement Program; BMI = body mass index; DVT = deep vein thrombosis  *P-value<0.05 from either a Wilcoxon-Mann Whitney test (continuous variables) or chi-square test (categorical variables).  ^a^Values presented as median (Q1-Q3) ; all others are presented as frequency (%).  ^b^At least 1 unit of packed or whole red blood cells given from the surgical start time up to and including 72 hours postoperatively.  ^c^Infection that occurs within 30 days after the operation and the infection involves only skin or subcutaneous tissue of the incision.  ^d^Preoperative evidence of a documented open wound at the time of the principal operative procedure. An open wound is a breach in the integrity of the skin or separation of skin edges and includes open surgical wounds, with or without cellulitis or purulent exudate. This does not include osteomyelitis or localized abscesses.  ^e^Infection that occurs within 30 days after the operation and the infection appears to be related to the operation and infection involved deep soft tissues (e.g., fascial and muscle layers) of the incision.  ^f^Readmission within 30 days was only available beginning in 2011; therefore, the percent provided reflects only the proportion of 2011-13 cases who were readmitted. | | | | |
